# Relationship between preoperative red blood cell distribution width and postoperative pneumonia in elderly patients with hip fracture: a retrospective cohort study

**DOI:** 10.1186/s13018-023-03732-6

**Published:** 2023-03-28

**Authors:** Daxue Zhang, Yu Zhang, Shiwei Yang, Lixin Sun, Ning Zhang, Shaocai Huang

**Affiliations:** 1 School of Nursing, Anhui Medical University, Hefei, China; 2 Department of Orthopedics, Zhejiang Hospital, Hangzhou, China; 3 Teaching Office, Shenzhen Second People’s Hospital, 3002 Sungang Road West, Futian District, Shenzhen City, 518000 China; 4 Department of Bone and Joint Bone Oncology, Shenzhen Second People’s Hospital, Shenzhen, China; 5 First Affiliated Hospital of Shenzhen University, Shenzhen, China

**Keywords:** Hip fracture, Red blood cell distribution width, Pneumonia, Saturation effect, Generalized additive model

## Abstract

**Objective:**

The relationship between the preoperative red blood cell distribution width and postoperative pneumonia in elderly patients with hip fractures remains unclear. This study investigated whether the preoperative red blood cell distribution width was associated with postoperative pneumonia in elderly patients with hip fractures.

**Methods:**

Clinical data of patients with hip fractures in the Department of orthopedics of a hospital from January 2012 to December 2021 were retrospectively analyzed. A generalized additive model was used to identify both linear and nonlinear relationships between red blood cell distribution width and postoperative pneumonia. A two-piecewise linear regression model was used to calculate the saturation effect. Subgroup analyses were performed using stratified logistic regression.

**Results:**

This study included a total of 1444 patients. The incidence of postoperative pneumonia was 6.30% (91/1444), the mean age of the patients was 77.55 ± 8.75 years, and 73.06% of them (1055/1444) were female. After full adjustment for covariates, the preoperative red blood cell distribution width showed a nonlinear relationship with postoperative pneumonia. The two-piecewise regression model showed an inflection point at 14.3%. On the left side of the inflection point, the incidence of postoperative pneumonia increased by 61% for every 1% increase in red blood cell distribution width (OR: 1.61, 95% CI 1.13–2.31, *P* = 0.0089). The effect size was not statistically significant on the right side of the inflection point (OR: 0.83, 95% CI 0.61–1.12, *P* = 0.2171).

**Conclusion:**

The relationship between preoperative red blood cell distribution width and incidence or postoperative pneumonia was nonlinear in elderly patients with hip fractures. The incidence of postoperative pneumonia was positively correlated with red blood cell distribution width when it was < 14.3%. A saturation effect was observed when the red blood cell distribution width reached 14.3%.

## Introduction

Hip fractures are becoming more frequent due to the increasing population of the elderly, and as a prevalent condition, its mortality and disability rates remain high [1]. According to a retrospective study [2] that examined hip fractures in urban Chinese adults, the incidence of hip fractures among those aged 55 years and older was 148.75 per 100,000, with the incidence in women being higher (180.72 per 100,000) than that in men (121.86 per 100,000). The incidence of hip fractures also increases with age. For elderly patients with hip fractures, surgery is the preferred treatment option, and the choice of an intramedullary nail or hip replacement depends on the type of fracture [3–5]. The mortality rate for patients with hip fractures remains as high as 17–27% within 1 year after surgery and more than 40% at 5 years postoperatively, despite ongoing advancements in medical care [6]. Pneumonia has been identified as the primary cause of postoperative death in elderly individuals with hip fractures [7, 8]. Postoperative pneumonia (POP) is a common complication of hip fracture in the elderly, with an incidence of 5.1–14.9% [9–11]. There are many risk factors for POP in patients with hip fractures; however, there is no shortage of contentious risk factors.

The red blood cell distribution width (RDW) is a measurement of variations in the size of red blood cells [12]. RDW is a reliable indicator of a disease under several conditions. RDW has been found to be associated with cardiovascular disease [13, 14] (e.g., stroke, atrial fibrillation, and heart failure), malignancy [15], and death [16, 17]. In recent years, RDW has been found to be an independent predictor of inflammatory and infectious diseases [18–20]. Elevated RDW has been reported to be associated with pulmonary infections after hip fracture surgery in the elderly; however, there are variations in available reports [21, 22].

To determine whether preoperative RDW and POP were related, we conducted a retrospective cohort study with Chinese patients with hip fracture as the study population.

## Methods

### Participants and methods

A total of 1444 patients were included in this retrospective cohort study. We collected clinical data on elderly patients with hip fracture who were `admitted to the orthopedic department of Shenzhen Second People's Hospital between January 2012 and December 2021. The collected clinical data included general patient demographic information, preoperative laboratory test results, intraoperative data, and short-term prognostic information.

**Fig. 1:**
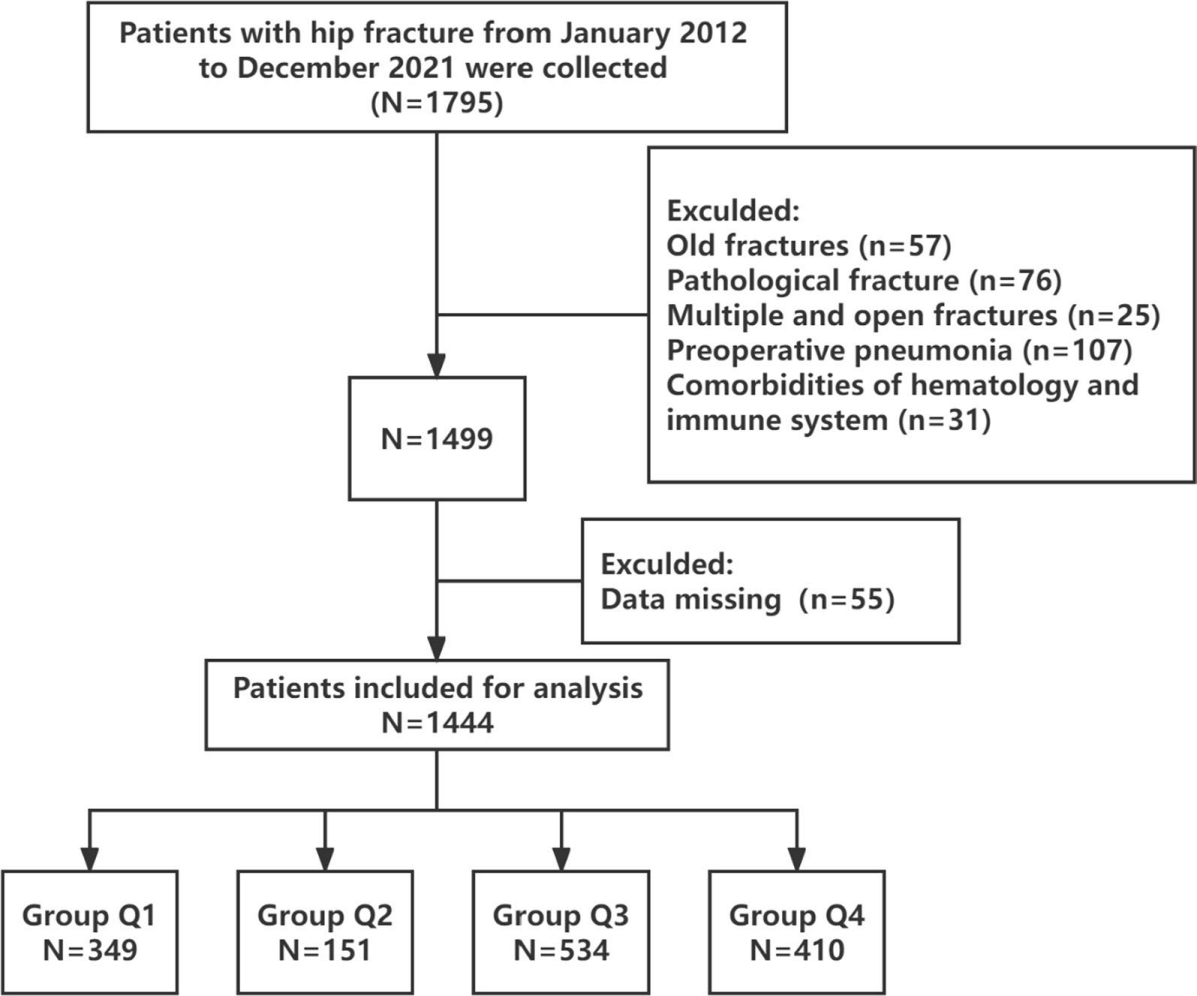
Flow chart of patient selection

Hip fractures with a confirmed radiographic diagnosis (including femoral neck or intertrochanteric fractures), age ≥ 60 years, and surgical intervention were included. Old fractures (time from injury to admission > 3 weeks), pathological fractures, periprosthetic fractures, multiple fractures, or open fractures, preoperatively diagnosed pulmonary infections, combined hematological and immune system diseases (like leukemia, rheumatoid arthritis, and systemic lupus erythematosus), and incomplete data were among the exclusion criteria. The Clinical Research Ethics Committee of Shenzhen Second People's Hospital approved this study, which was conducted in compliance with the Declaration of Helsinki and medical ethics guidelines (20,210,620,213,357,012-FS01). The data were retrospective and anonymous; therefore, the requirement for informed consent was waived. See the flowchart for details (Fig. 1).

### Clinical variables and outcomes

The RDW at admission served as the independent variable and the incidence of POP served as the dependent variable. Covariates included demographic data [e.g., age, sex, body mass index (BMI), classification of fracture, etc.], comorbidities [e.g., hypertension, diabetes mellitus, coronary artery disease, stroke, chronic obstructive pulmonary disease (COPD), etc.], preoperative laboratory indices (e.g., hemoglobin, leukocyte count, neutrophil count, monocyte count, albumin, globulin, blood urea nitrogen, and serum creatinine), surgery-related variables [operation duration, anesthesia methods**,** operation methods, American Society of Anesthesiologists (ASA) classification, intraoperative blood loss, etc.], and short-term prognostic information [e.g., length of hospital stay, intensive care unit (ICU) admission, and in-hospital death].

### Definition of pneumonia

The diagnosis of pneumonia was based on the American Thoracic Society guidelines for healthcare-associated pneumonia [23] and Guidelines for the Diagnosis and Treatment of Adult hospital-acquired pneumonia and ventilator-associated pneumonia in China (2018 edition) [24]: (1) newly developed cough, sputum production, or aggravation of symptoms of original respiratory disease and purulent sputum with or without chest pain after admission; (2) fever; (3) physical examination showing signs of lung consolidation and/or wet rales; (4) white blood cell count > 10 × 10^9^/L or < 4 × 10^9^/L; and (5) chest X-ray film showing patchy infiltrating shadow or interstitial changes. If a patient had any of the above items 1–4 plus item 5 and did not have other pulmonary diseases, such as tuberculosis, lung cancer, or pulmonary embolism, pneumonia was diagnosed. POP was used as the study outcome indicator from the first 24 h after surgery until discharge.

### Statistical analysis

Continuous variables are expressed as mean ± standard deviation (normal distribution) or median (interquartile range) (skewed distribution), and categorical variables are expressed as frequencies or percentages.

One-way analysis of variance (ANOVA) (normal distribution), Kruskal–Wallis H (skewed distribution) test, and chi-square test (categorical variables) were used to determine any significant differences among the groups according to the RDW quartiles. To group the RDW in quartiles, the RDW data were ordered from smallest to largest and the ordered distribution was divided into four parts of equal size. Patients were classified into four groups according to quartiles of RDW, and baseline characteristics were compared among the groups. Second, univariate analysis with logistic regression was performed to evaluate the association between variables and POP. The results are expressed as the effect size with odds ratios (OR) and 95% confidence intervals (CI). Statistical analysis consisted of three main steps.

Step 1: Univariate and multivariate binary logistic regression models were used. We constructed three distinct models: a non-adjusted model (with unadjusted covariates), a minimally adjusted model (with adjusted sociodemographic variables only), and a fully adjusted model (with adjusted covariates, as shown in Table 1). Variance Infusion Factors > 10 were removed from the fully adjusted model. Step 2: Considering that logistic regression cannot handle the nonlinear relationship and the possibility of a nonlinear relationship between RDW and the incidence of POP cannot be ruled out, smooth curve fitting (penalized spline method) was used to address nonlinearity. We used a two-piecewise linear regression model to examine the saturation effect of RDW on the incidence of POP. The inflection point for the RDW was determined using “exploratory” analyses, which involved moving the trial inflection point along the predefined interval and picking up the one that gave the maximum model likelihood. We also performed a log-likelihood ratio test and compared the one-line linear regression model with a two-piecewise linear model. Step 3: To ensure the robustness of the data analysis, we converted RDW into a categorical variable based on quartiles for sensitivity analysis to verify the results of RDW as a continuous variable.

**Table 1:** Baseline characteristics of the 1444 participants

RDW (%)	Q1 (12.00–12.30)	Q2 (12.60–12.80)	Q3 (13.00–13.37)	Q4 (14.00–15.40)	*P*-value
N	349	151	534	410	
Age (years)	75.17 (8.88)	75.85 (8.75)	77.70 (8.62)	79.98 (8.08)	< 0.001
Sex (n, %)					0.033
Male	75 (21.49%)	48 (31.79%)	144 (26.97%)	122 (29.76%)	
Female	274 (78.51%)	103 (68.21%)	390 (73.03%)	288 (70.24%)	
BMI (kg/m^2^)	22.12 (3.14)	22.41 (3.13)	22.09 (3.38)	21.69 (3.21)	0.074
Classification of fracture (n, %)					< 0.001
Intertrochanteric fracture	255 (73.07%)	100 (66.23%)	339 (63.48%)	231 (56.34%)	
Femoral neck fracture	94 (26.93%)	51 (33.77%)	195 (36.52%)	179 (43.66%)	
Time from fracture to surgery (hours)	76.00 (50.00–141.00)	72.00 (47.00–127.50)	76.50 (49.00–137.75)	90.00 (60.00–144.75)	0.023
Comorbidity	234 (67.05%)	106 (70.20%)	392 (73.41%)	310 (75.61%)	0.053
Smoking status (n, %)	14 (4.01%)	8 (5.30%)	23 (4.31%)	22 (5.37%)	0.782
COPD (n, %)	4 (1.15%)	6 (3.97%)	12 (2.25%)	7 (1.71%)	0.201
Asthma (n, %)	6 (1.72%)	0 (0.00%)	6 (1.12%)	9 (2.20%)	0.224
Hypertension (n, %)	142 (40.69%)	67 (44.37%)	264 (49.44%)	221 (53.90%)	0.002
Coronary heart disease (n, %)	36 (10.32%)	13 (8.61%)	72 (13.48%)	69 (16.83%)	0.018
Atrial fibrillation (n, %)	12 (3.44%)	9 (5.96%)	24 (4.49%)	25 (6.10%)	0.328
Stroke (n, %)	54 (15.47%)	28 (18.54%)	100 (18.73%)	75 (18.29%)	0.630
Parkinson’s disease (n, %)	8 (2.29%)	4 (2.65%)	16 (3.00%)	5 (1.22%)	0.336
Diabetes mellitus (n, %)	80 (22.92%)	36 (23.84%)	125 (23.41%)	85 (20.73%)	0.760
WBC count (× 10^9/L)	9.04 (2.96)	9.87 (3.35)	9.11 (2.93)	9.19 (3.11)	0.033
Lymphocyte count (× 10^9/L)	1.26 (0.50)	1.29 (0.59)	1.32 (0.65)	1.28 (0.59)	0.458
Neutrophil count (× 10^9/L)	7.07 (2.96)	7.95 (3.34)	7.12 (2.76)	7.33 (4.40)	0.038
Monocyte count (× 10^9/L)	0.55 (0.41–0.72)	0.59 (0.45–0.75)	0.53 (0.40–0.70)	0.53 (0.39–0.71)	0.045
MLR	0.47 (0.33–0.66)	0.49 (0.37–0.66)	0.44 (0.31–0.61)	0.44 (0.30–0.63)	0.051
NLR	5.48 (3.63–8.27)	6.10 (4.09–9.32)	5.27 (3.68–7.96)	5.59 (3.91–8.20)	0.196
Platelet count (× 10^9/L)	211.27 (74.57)	206.82 (80.06)	200.96 (64.76)	215.03 (89.14)	0.034
Hemoglobin (g/L)	121.00 (16.40)	120.82 (16.69)	118.38 (18.03)	108.76 (20.04)	< 0.001
Albumin (g/L)	39.01 (4.06)	38.69 (4.04)	38.07 (4.11)	36.99 (5.05)	< 0.001
Globulin (g/L)	28.55 (5.27)	30.12 (16.65)	27.86 (4.55)	28.62 (5.62)	0.008
AGR	1.41 (0.30)	1.36 (0.24)	1.40 (0.28)	1.33 (0.29)	< 0.001
Serum creatinine (umol/L)	60.00 (48.40–73.10)	65.30 (54.10–76.95)	66.00 (53.85–82.55)	67.45 (55.05–88.27)	< 0.001
Blood urea nitrogen (mmol/L)	4.50 (2.84–6.30)	4.90 (3.33–6.42)	5.10 (3.27–6.70)	5.30 (3.61–7.40)	< 0.001
Operation method (n, %)					0.110
Internal fixation	128 (36.68%)	71 (47.02%)	221 (41.39%)	179 (43.66%)	
Hip replacement	221 (63.32%)	80 (52.98%)	313 (58.61%)	231 (56.34%)	
Anesthesia method (n, %)					0.604
General anesthesia	266 (76.22%)	111 (73.51%)	416 (77.90%)	307 (74.88%)	
Non-general anesthesia	83 (23.78%)	40 (26.49%)	118 (22.10%)	103 (25.12%)	
ASA classification (n, %)					< 0.001
≤ 2	186 (53.30%)	79 (52.32%)	231 (43.26%)	128 (31.22%)	
≥ 3	163 (46.70%)	72 (47.68%)	303 (56.74%)	282 (68.78%)	
Intraoperative blood loss (mL)	200.00 (100.00–300.00)	200.00 (100.00–300.00)	200.00 (100.00–300.00)	200.00 (100.00–300.00)	0.153
Operation duration (min)	84.22 (31.99)	91.04 (32.70)	85.25 (35.76)	83.00 (39.70)	0.123
In-hospital death (n, %)	0 (0.00%)	1 (0.66%)	3 (0.56%)	1 (0.24%)	0.477
ICU admission (n, %)	6 (1.72%)	1 (0.66%)	13 (2.43%)	11 (2.68%)	0.447
POP (n, %)	14 (4.01%)	4 (2.65%)	31 (5.81%)	42 (10.24%)	< 0.001
Length of hospital stay (days)	10.00 (8.00–14.00)	10.00 (7.00–13.00)	10.00 (8.00–15.00)	11.00 (8.00–17.00)	0.025

*RDW* Red blood cell distribution width; *BMI* Body mass index; *COPD* Chronic obstructive pulmonary disease; *WBC* White blood cell; *MLR* Monocyte lymphocyte ratio; *NLR* Neutrophil lymphocyte ratio; *AGR* Albumin–globulin ratio; *ASA* American Society of Anesthesiologists; *ICU* Intensive care unit; *POP* Postoperative pneumonia

Analyses were performed using the statistical software packages R (http://www.R-project.org, The R Foundation) and EmpowerStats (http://www.empowerstats.com, X&Y Solutions, Inc, Boston, MA, USA). *P* values < 0.05 (two-sided) were considered statistically significant.

## Results

### Baseline characteristics of participants

The baseline characteristics of the participants is shown in Table 1. We divided RDW into four groups (Q1–Q4) based on the quartile principle. The mean age of the 1444 patients was 77.55 ± 8.75 years; among them, 73.06% (1055/1444) were female and the incidence of POP was 6.30% (91/1444). Compared with the Q1–Q3 group, patients in the Q4 group had a higher mean age, longer time from fracture to surgery, and length of hospital stay; higher levels of platelet count, serum creatinine, and blood urea nitrogen; and a higher percentage of POP, femoral neck fracture, coronary artery disease, hypertension, and ASA classification ≥ 3 (*P* < 0.05). However, patients had a lower monocyte count, hemoglobin, albumin, and albumin–globulin ratio (AGR) (*P* < 0.05).

### Univariate analysis

Univariate analysis showed that age, sex, comorbidity, coronary heart disease, stroke, Parkinson’s disease, white blood cell (WBC) count, neutrophil count, monocyte lymphocyte ratio (MLR), neutrophil lymphocyte ratio (NLR), hemoglobin, albumin, AGR, serum creatinine, blood urea nitrogen, ASA classification, intraoperative blood loss, ICU admission, and length of hospital stay were positively correlated with the incidence of POP (Table 2).

**Table 2:** Univariate analysis of postoperative pneumonia

	Statistics	OR (95% CI)	*P*-value
Age (years)	77.54 ± 8.73	1.08 (1.05, 1.10)	< 0.0001
Sex (n, %)			
Male	389 (26.94%)	1.0	
Female	1055 (73.06%)	0.63 (0.40, 0.98)	0.0398
BMI (kg/m^2^)	22.02 ± 3.25	0.97 (0.91, 1.04)	0.3815
Classification of fracture (n, %)			
Intertrochanteric fracture	925 (64.06%)	1.0	
Femoral neck fracture	519 (35.94%)	1.24 (0.80, 1.91)	0.3334
Time from fracture to surgery (hours)	125.38 ± 262.26	1.00 (1.00, 1.00)	0.5260
Comorbidity	1042 (72.16%)	2.03 (1.15, 3.58)	0.0143
Smoking status (n, %)	67 (4.64%)	1.80 (0.80, 4.05)	0.1583
COPD (n, %)	29 (2.01%)	2.44 (0.83, 7.17)	0.1043
Asthma (n, %)	21 (1.45%)	0.74 (0.10, 5.58)	0.7707
Hypertension (n, %)	694 (48.06%)	1.28 (0.84, 1.96)	0.2548
Coronary heart disease (n, %)	190 (13.16%)	2.10 (1.26, 3.51)	0.0046
Atrial fibrillation (n, %)	70 (4.85%)	1.71 (0.76, 3.84)	0.1968
Stroke (n, %)	257 (17.80%)	2.31 (1.45, 3.67)	0.0004
Parkinson’s disease (n, %)	33 (2.29%)	2.75 (1.04, 7.30)	0.0422
Diabetes mellitus (n, %)	326 (22.58%)	1.25 (0.77, 2.02)	0.3716
WBC count (× 10^9/L)	9.20 ± 3.04	1.10 (1.03, 1.16)	0.0034
Lymphocyte count (× 10^9/L)	1.29 ± 0.59	0.68 (0.44, 1.05)	0.0816
Neutrophil count (× 10^9/L)	7.25 ± 3.41	1.06 (1.01, 1.11)	0.0178
Monocyte count (× 10^9/L)	0.59 ± 0.31	1.07 (0.56, 2.04)	0.8456
MLR	0.53 ± 0.41	1.47 (1.04, 2.09)	0.0309
NLR	6.94 ± 5.76	1.04 (1.01, 1.07)	0.0037
Platelet count (× 10^9/L)	208.06 ± 76.44	1.00 (1.00, 1.00)	0.8809
Hemoglobin (g/L)	116.54 ± 18.79	0.98 (0.97, 0.99)	< 0.0001
Albumin (g/L)	38.06 ± 4.44	0.92 (0.88, 0.96)	< 0.0001
Globulin (g/L)	28.48 ± 7.25	1.02 (1.00, 1.04)	0.0503
AGR	1.38 ± 0.28	0.11 (0.05, 0.26)	< 0.0001
Serum creatinine (umol/L)	81.17 ± 82.19	1.00 (1.00, 1.00)	0.0174
Blood urea nitrogen (mmol/L)	5.60 ± 3.75	1.06 (1.01, 1.10)	0.0092
Operation method (n, %)			
Internal fixation	599 (41.48%)	1.0	
Hip replacement	845 (58.52%)	1.33 (0.85, 2.07)	0.2077
Anesthesia method (n, %)			
General anesthesia	1100 (76.18%)	1.0	
Non-general anesthesia	344 (23.82%)	1.02 (0.62, 1.68)	0.9349
ASA classification (n, %)			
≤ 2	624 (43.21%)	1.0	
≥ 3	820 (56.79%)	2.10 (1.31, 3.36)	0.0021
Operation duration (min)	84.97 ± 35.81	0.99 (0.99, 1.00)	0.1428
Intraoperative blood loss (mL)	246.46 ± 195.38	1.00 (1.00, 1.00)	0.0170
In-hospital death (n, %)	5 (0.35%)	3.75 (0.41, 33.88)	0.2396
ICU admission (n, %)	31 (2.15%)	14.29 (6.79, 30.06)	< 0.0001
Length of hospital stay (days)	12.12 ± 6.91	1.09 (1. 07, 1.12)	< 0.0001

*BMI* Body mass index; *COPD* Chronic obstructive pulmonary disease; *WBC* White blood cell; *MLR* Monocyte lymphocyte ratio; *NLR* Neutrophil lymphocyte ratio; *AGR* Albumin–globulin ratio; *ASA* American Society of Anesthesiologists; *ICU* Intensive care unit

### Results of the relationship between RDW and POP

Three models were used to evaluate the relationship between RDW and POP (Table 3). In the unadjusted model, each 1% increase in RDW was associated with a 32% increase in the incidence of POP (OR, 1.32; 95% CI 1.15–1.51, *P* < 0.0001). In the minimally adjusted model for age and sex, an association was also identified (OR, 1.21; 95% CI 1.05–1.41; *P* = 0.0076). However, in adjusted model II, we did not find a linear relationship between RDW and POP (*P* = 0.2354).

**Table 3:** Relationship between RDW and POP in different models

Exposure	Non-adjusted *P*-value	Adjust I *P*-value	Adjust II *P*-value
RDW	1.32 (1.15, 1.51) < 0.0001	1.21 (1.05, 1.40) 0.0076	1.10 (0.94, 1.30) 0.2354
RDW quartile			
Q1	1.0	1.0	1.0
Q2	0.65 (0.21, 2.01) 0.4559	0.59 (0.19, 1.85) 0.3692	0.54 (0.17, 1.74) 0.2996
Q3	1.47 (0.77, 2.81) 0.2386	1.23 (0.64, 2.37) 0.5320	1.20 (0.60, 2.39) 0.6005
Q4	2.73 (1.47, 5.09) 0.0016	2.00 (1.06, 3.79) 0.0322	1.68 (0.84, 3.35) 0.1432
*P* for trend	0.0004	0.0104	0.0670

Non-adjusted model adjusted for: None

Adjust I model adjusted for age, sex

Adjust II model adjusted for age, sex, comorbidity, coronary heart disease, Stroke, Parkinson’s disease, WBC count, MLR, NLR, hemoglobin, albumin, AGR, serum creatinine, blood urea nitrogen, ASA classification, intraoperative blood loss, ICU admission

For sensitivity analysis, we divided RDW into four groups, and the results showed the same increasing trend in the incidence of POP in the unadjusted and minimally adjusted models (All *P* < 0.05), but the same trend was not found in adjusted model II (*P* = 0.0670).

### Results of nonlinear association between RDW and POP

**Fig. 2:**
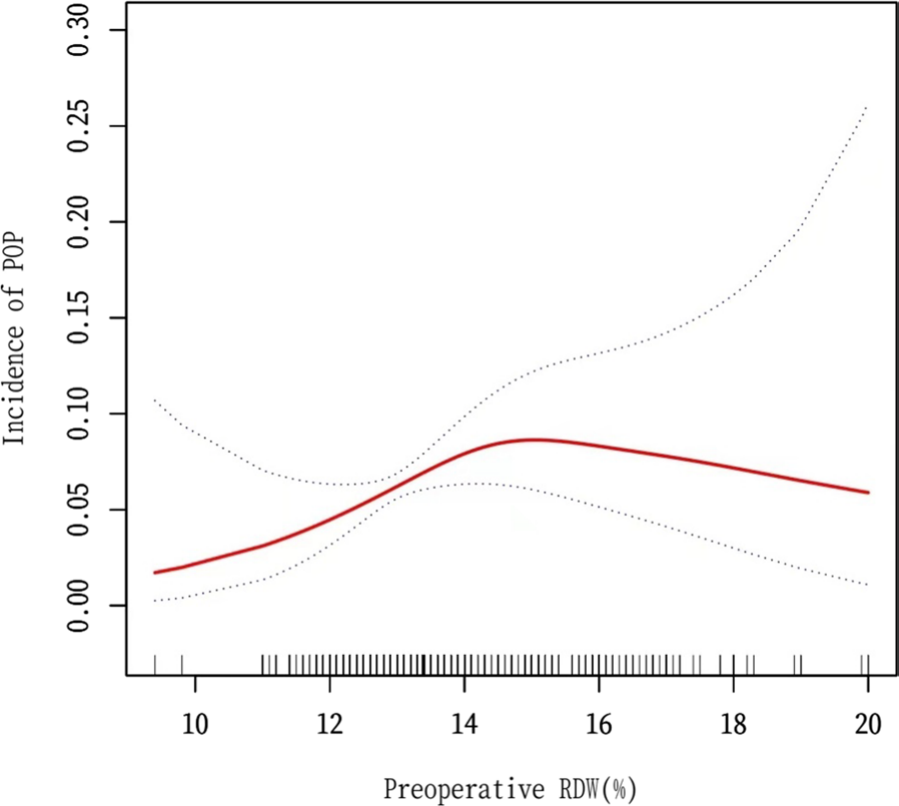
The correlation between RDW and POP in patients with hip fracture. Adjust: age, sex, comorbidity, coronary heart disease, stroke, Parkinson’s disease, WBC count, MLR, NLR, hemoglobin, albumin, AGR, serum creatinine, blood urea nitrogen, ASA classification, intraoperative blood loss, ICU admission

As shown in the smoothing spline, the RDW was found to have a nonlinear relationship with POP (Fig. 2). The two-piecewise regression model showed that the inflection point was 14.3% after adjusting for covariates (age, sex, comorbidity, coronary heart disease, stroke, Parkinson’s disease, WBC count, MLR, NLR, hemoglobin, albumin, AGR, serum creatinine, blood urea nitrogen, ASA classification, intraoperative blood loss, and ICU admission). On the left side of the inflection point, the incidence increased by 61% per 1% increase in RDW (95% CI 1.13–2.31, *P* = 0.0089 (Table 4). The effect size was not statistically significant on the right side of the inflection point (OR,0.83; 95% CI 0.61–1.12, *P* = 0.2171).

**Table 4:** Nonlinearity explanation of RDW and POP using the two-phase linear model

	Incidence of POP OR ( 95% CI) *P*-value
*Model I*	
One line effect	1.10 (0.94, 1.30) 0.2354
*Model II*	
Fitting model using a two-piecewise linear model	
Inflection point	14.3%
< Inflection point	1.61 (1.13, 2.31) 0.0089
> Inflection point	0.83 (0.61, 1.12) 0.2171
*P* for log likelihood ratio test	0.015

Effect: POP, Cause: RDW

Adjusted for age, sex, comorbidity, coronary heart disease, stroke, Parkinson’s disease, WBC count, MLR, NLR, Hemoglobin, Albumin, AGR, serum creatinine, blood urea nitrogen, ASA classification, intraoperative blood loss, and ICU admission

### Results of subgroup analyses

We did not find any statistically significant interactions between age, sex, comorbidity, coronary heart disease, stroke, Parkinson’s disease, WBC count, NLR, MLR, hemoglobin, albumin, AGR, serum creatinine, blood urea nitrogen, ASA classification, intraoperative blood loss, and ICU admission (Table 5).

**Table 5:** Effect size of preoperative RDW on POP in prespecified and exploratory subgroups

Characteristics	No. of patients	OR (95% CI)	*P* for interaction
Age			0.6675
60–73	472	1.45 (1.02, 2.05)	
74–81	457	1.18 (0.86, 1.61)	
82–108	515	1.23 (1.04, 1.46)	
Sex			0.7661
Male	389	1.28 (1.02, 1.60)	
Female	1055	1.33 (1.13, 1.57)	
Comorbidity	1042	1.29 (1.12, 1.50)	0.8063
Coronary heart disease	190	1.41 (1.07, 1.85)	0.5415
Stroke	257	1.52 (1.17, 1.97)	0.2313
Parkinson’s disease	33	1.14 (0.31, 4.20)	0.8137
WBC count			0.1311
≤ 7.99	464	1.42 (1.12, 1.81)	
8.00–9.99	394	1.54 (1.20, 1.97)	
≥ 10.00	586	1.12 (0.90, 1.40)	
MLR			0.0967
≤ 0.366	480	1.59 (1.29, 1.97)	
0.367–0.566	482	1.17 (0.88, 1.55)	
≥ 0.567	482	1.18 (0.94, 1.48)	
NLR			0.7724
≤ 4.32	481	1.43 (1.10, 1.87)	
4.33–6.99	480	1.29 (1.02, 1.64)	
≥ 7.00	483	1.27 (1.04, 1.55)	
Hemoglobin			0.1695
≤ 108	464	1.28 (1.07, 1.53)	
108–124	473	1.01 (0.71, 1.43)	
≥ 125	507	1.59 (1.15, 2.20)	
Albumin			0.4294
≤ 37.0	441	1.25 (1.03, 1.52)	
37.1–39.4	515	1.14 (0.84, 1.56)	
≥ 39.5	488	1.47 (1.14, 1.89)	
AGR			0.6146
≤ 1.29	481	1.24 (1.05, 1.47)	
1.30–1.42	481	1.42 (1.07, 1.90)	
≥ 1.43	482	1.14 (0.79, 1.66)	
Serum creatinine			0.9036
≤ 56.9	480	1.28 (0.97, 1.68)	
57–73.8	480	1.27 (0.99, 1.62)	
≥ 73.9	484	1.35 (1.10, 1.66)	
Blood urea nitrogen			0.2690
≤ 3.81	475	1.09 (0.81, 1.48)	
3.82–6.19	481	1.50 (1.17, 1.93)	
≥ 6.20	488	1.31 (1.07, 1.60)	
ASA classification			0.3382
≤ 2	624	1.43 (1.12, 1.84)	
≥ 3	820	1.24 (1.05, 1.45)	
Intraoperative blood loss			0.4680
≤ 149	471	1.19 (0.95, 1.48)	
150–299	420	1.43 (1.15, 1.79)	
≥ 300	553	1.37 (1.04, 1.81)	
ICU admission	31	1.58 (0.93, 2.66)	0.3819

*WBC* White blood cell; *MLR* Monocyte lymphocyte ratio; *NLR* Neutrophil lymphocyte ratio; *AGR* Albumin–globulin ratio; *ASA* American Society of Anesthesiologists; *ICU* Intensive care unit

## Discussion

In this study, we used a generalized linear model to investigate the association between preoperative RDW and POP in elderly patients with hip fractures who were treated at the Department of Orthopedics of the Second People's Hospital of Shenzhen over a 10-year period. A nonlinear association between preoperative RDW and the incidence of postoperative pneumonia was discovered after 1444 patients' data were analyzed. To our knowledge, no previous research has mentioned this link. We found different correlations on both sides of the inflection point. On the left side of the inflection point, RDW was positively correlated with the incidence of POP (OR: 1.61, 95% CI 1.13–2.31, *P* = 0.0089), while on the right side of the inflection point, the effect seemed to be saturated with no statistical significance (OR, 0.83; 95% CI 0.61–1.12; *P* = 0.2171). Although medical technology has made great progress, there are still many problems associated with hip fractures, such as the decline in body function [25] and the occurrence of pneumonia.

Pneumonia, a common postoperative complication in elderly patients with hip fractures [9, 10, 26], causes prolonged hospitalization, increases cost and mortality, and increases the risk of readmission [27, 28], which greatly drains social resources and is a burden to patients' lives. Therefore, clinical care providers are required to take measures to reduce the incidence of POP. Numerous studies have identified risk factors for POP. Although some of these studies have identified RDW as a risk factor for POP [21], the fundamental relationship is still difficult to establish. Through this study, we have not only shown that preoperative RDW is a risk factor for POP but also pinpointed the inflection point, and this would give clinical healthcare practitioners a reference point on which to base future actions.

RDW was initially used to differentiate between anemia and other conditions [29], and a recent study demonstrated that increased RDW is a sign of an inflammatory response [30]. Preoperative RDW was demonstrated to be a significant predictor of POP in a study on the risk factors for pneumonia following meningioma excision [31]. Similarly, in a study of POP in patients with hip fracture [22], preoperative RDW was found to have a significant impact in predicting the development of pneumonia following hip fracture surgery. Additionally, another study [32] found that in patients undergoing coronary artery bypass grafting, the incidence of postoperative pneumonia increased from 1.5 to 4.1% in patients with RDW ≥ 12.36% compared to that of those with RDW < 11.72%, with a relative risk (*RR*) of 2.72 when unadjusted for variables (95% CI 1.37–5.44, *P* = 0.004), which remained statistically significant after multifactorial correction (*RR*:1.95, 95% CI 1.02–3.75, *P* = 0.044). Consistent with these results, our study also showed a similarly increased risk of POP in elderly patients with hip fractures with increasing RDW in the unadjusted model (OR: 1.32, 95% CI 1.15–1.51, *P* < 0.0001). Therefore, our study confirms that preoperative RDW is associated with POP. Clinical staffs can increase their attention to RDW for early assessment of patients at high risk of developing pneumonia.

The findings of this study demonstrated a correlation between preoperative RDW and POP incidence. Increased production of pro-inflammatory cytokines may be the cause of the inflammatory response that leads to poor maturation of peripheral blood erythrocytes and an increase in naïve erythrocytes, which in turn contributes to the increase in RDW [33]. Previous studies [32] have shown that RDW is associated with the risk of postoperative inflammatory complications in patients. RDW is a valuable and sensitive marker of high levels of inflammatory activity in patients with community-acquired pneumonia [34]. RDW is a straightforward and low-cost metric that reflects the degree of red blood cell volume heterogeneity. Because less deformable red blood cells worsen tissue oxygenation, elevated RDW may be associated with worse prognosis [29]. However, the relationship between RDW and pneumonia remains unclear. RDW is connected to interleukin-6 (IL-6), C-reactive protein, and erythrocyte sedimentation rate, indicating that RDW levels may be a measure of inflammation, which is one of the mechanisms that has been considered [35–37]. Elevated RDW levels suggest that patients may develop organ dysfunction, which could have a negative impact on their outcomes [38, 39]. Chen et al. [21] retrospectively analyzed clinical data from elderly patients with hip fractures and found that the risk of developing POP was 5.097 times higher for patients with RDW > 14.8% than for patients with RDW < 14.8% (95% CI 2.036–12.759, *P* = 0.001). Another study found the same association between RDW in patients with coronavirus disease 2019 (COVID-19) and their mortality and disease severity [33, 40], with higher levels of RDW being associated with poorer outcomes in patients with COVID-19 and higher RDW in non-survivors and patients with more severe symptoms than in survivors and patients with less severe symptoms. However, Zhao et al. [41] found that preoperative RDW was not a risk factor for POP in a study of 1495 elderly patients (> 65 years of age) who underwent surgery for femoral intertrochanteric fractures. Considering that such a problem exists because of the inconsistent population studied, we conducted a retrospective cohort study adjusted for relevant variables and used a generalized additive model (GAM) to explore the relationship between preoperative RDW and POP, which was determined to be associated with POP by smoothed curve fitting.

However, our study did not find any correlation between BMI, smoking status, and POP. In contrast, Xiang [11] and Gao [42] demonstrated that BMI and smoking were independent risk factors for POP. There was no correlation between smoking and pneumonia due to the limitations of the retrospective study, in which the prevalence of smoking in patients was much lower than in the actual situation; moreover, our study population was predominantly female. Therefore, additional prospective studies should be conducted to determine whether smoking is an independent risk factor of POP.

This study has several advantages. It utilizes the GAM to elucidate the nonlinear relationship between RDW and POP. The use of GAM fits a smoothing spline and helps in understanding the real relationship between variables and outcomes. Rigorous statistical adjustment was performed for confounding factors owing to the retrospective nature of the study. Interaction analysis also improves the reliability of the data. Notably, the study found that when the preoperative RDW was < 14.3%, the incidence of POP increased by 61% for every 1% increase in RDW. In clinical practice, this association can be used as a predictor of POP risk. Therefore, the size of the preoperative RDW can be used to determine whether appropriate preventive measures, such as the employment of a dedicated ortho-geriatrician in an orthopedic department, allow for the optimization of patients’ clinical conditions [43].

### Limitations

This study has some limitations. The evidence for an association between exposure and outcome was weaker in this study than in previous prospective studies. In addition, this study was conducted in a population aged > 60 years; therefore, future studies should be conducted in younger and middle-aged populations to determine the correlation between preoperative RDW and POP. This was an observational study; therefore, it was inevitably subject to confounding factors. Hence, we could only adjust for measurable confounders but not for unmeasurable ones. For example, our study did not collect information on whether the patients had consumed alcohol [44] or previously had pneumonia; furthermore, preoperative laboratory markers (e.g., C-reactive protein and erythrocyte sedimentation rate were not collected [45, 46]). These variables are risk factors for POP, and could have affected the accuracy of our results. Therefore, prospective studies with larger sample sizes and more variables are required to validate the relationship between preoperative RDW and POP. Finally, patients with preoperative pneumonia were excluded from this study. Preoperative pneumonia has been found to be associated with the risk of postoperative adverse events in patients with hip fractures [47, 48]. Therefore, future studies on preoperative pneumonia in elderly patients with hip fracture should be conducted to determine whether RDW and preoperative pneumonia have the same trend as POP.

## Conclusion

There was a nonlinear relationship between preoperative RDW and the incidence of POP in elderly patients with hip fractures. The incidence of POP was positively correlated with the RDW when the RDW was below 14.3%; however, no correlation was found when the RDW reached 14.3%.

## Data Availability

The datasets used or analyzed during the current study are available from the Corresponding author upon reasonable request.

## Abbreviations

OR: Odds ratio
CI: Confidence interval
POP: Postoperative pneumonia
RDW: Red blood cell distribution width
BMI: Body mass index
ICU: Intensive care unit
COPD: Chronic obstructive pulmonary disease
WBC: White blood cell
MLR: Monocytes lymphocyte ratio
NLR: Neutrophil lymphocyte ratio
AGR: Albumin–globulin ratio
ASA: American Society of Anesthesiologists
RR: Relative risk
COVID-19: Coronavirus disease 2019
GAM: Generalized additive model
IL: Interleukin
